# The worsening effect of anemia on left ventricular function and global strain in type 2 diabetes mellitus patients: a 3.0 T CMR feature tracking study

**DOI:** 10.1186/s12933-023-01745-3

**Published:** 2023-01-24

**Authors:** Wen-Lei Qian, Rong Xu, Rui Shi, Yuan Li, Ying-Kun Guo, Han Fang, Li jiang, Zhi-Gang Yang

**Affiliations:** 1 Department of Radiology, West China Hospital, Sichuan University, 37# Guo Xue Xiang, Chengdu, 610041 Sichuan China; 2 Department of Radiology, Key Laboratory of Obstetric & Gynecologic and Pediatric Diseases and Birth Defects of Ministry of Education, West China Second University Hospital, Sichuan University, 20# South Renmin Road, Chengdu, 610041 Sichuan China

**Keywords:** Type 2 diabetes mellitus, Anemia, Hemoglobin, Cardiac magnetic resonance, Feature tracking, Left ventricular function

## Abstract

**Objective:**

To explore the additive effects of anemia on left ventricular (LV) global strains in patients with type 2 diabetes mellitus (T2DM) with or without anemia via cardiac magnetic resonance (CMR) feature tracking technology.

**Materials and methods:**

236 T2DM patients with or without anemia and 67 controls who underwent CMR examination were retrospectively enrolled. LV function parameters, LV global radial peak strain (GRPS), longitudinal peak strain (GLPS), and circumferential peak strain (GCPS) were used to analyze the function and global strain of the heart. One-way analysis of variance and the chi-square test were used for intergroup analysis. Multivariable linear regression analysis was performed for the two T2DM groups to explore factors associated with LV global strains.

**Results:**

The T2DM group with anemia was oldest and had a lowest hemoglobin (Hb) concentration, lowest estimated glomerular filtration rate, highest LV end-systolic volume index, highest end-diastolic volume index and highest LV mass index than the control group and T2DM without anemia group (all P ≤ 0.001). Besides, The LV global peak strains in all three directions worsened successively from the control group to the T2DM without anemia group to the T2DM with anemia group (all p < 0.001). Among all clinical indices, the decrease in Hb was independently associated with the worsening in GRPS (β = 0.237, p = 0.001), GCPS (β = 0.326, p < 0.001), and GLPS (β = 0.265, p < 0.001).

**Conclusion:**

Anemia has additive deleterious effects on LV function and LV global strains in patients with T2DM. Regular detection and early intervention of anemia might be beneficial for T2DM patients.

## Introduction

Type 2 diabetes mellitus (T2DM) is a chronic and systemic metabolic disease characterized by resistance to insulin or insufficient production of it. According to the new International Diabetes Federation (IDF) Diabetes Atlas, 536.6 million people suffered from diabetes in 2021, and diabetes-related health expenditures were estimated to be 966 billion USD in all IDF regions [1]. Cardiovascular diseases have become the leading cause of the world’s deaths, while diabetes could double the risks of many cardiovascular diseases independently and was estimated to be responsible for 11% of cardiovascular deaths [2]. The red blood cell (RBC) count of patients with anemia is insufficient to meet physiological needs [3]. Anemia patients are suffered from the lack of oxygen supply and some damages occur in the cardiovascular system [4]. Some studies have proven that anemia is a risk factor of adverse cardiovascular outcomes, such as heart failure and increased mortality [5, 6].

T2DM and anemia can each damage the cardiovascular system in their own ways [4, 7, 8]. Furthermore, cardiac function seemed to worsen when they attack the heart synergistically [9]. Many other risk factors which would further damage heart when combined with T2DM, such as hypertension, obesity and hyperlipemia [10–13] have been well explored. However, anemia is common but often neglected in patients with T2DM [14]. Thus, the synergistic effects of T2DM and anemia on the heart may be underestimated and insufficiently explored.

Cardiac magnetic resonance (CMR) is an important imaging modality for cardiology due to the unique and complex imaging techniques involved. In addition, CMR could utilize myocardial feature tracking technology to reveal cardiac function and potential incipient stage damage [15]. A previous study using echocardiography have reported the result that the heart diastolic dysfunction of T2DM patients is associated with anemia. However, to the best of our knowledge, no study has used CMR feature tracking technology to explore the combined effects of T2DM and anemia on the heart. Thus, our study uses the CMR feature tracking technology to explore the additive effects of anemia on LV function and LV strain in patients with T2DM, which could help doctors to better understand and manage T2DM patients with anemia.

## Methods

### Study population

This study protocol was approved by the Biomedical Research Ethics Committee of our hospital. Informed consent was waived due to the retrospective nature of the research.

**Fig. 1:**
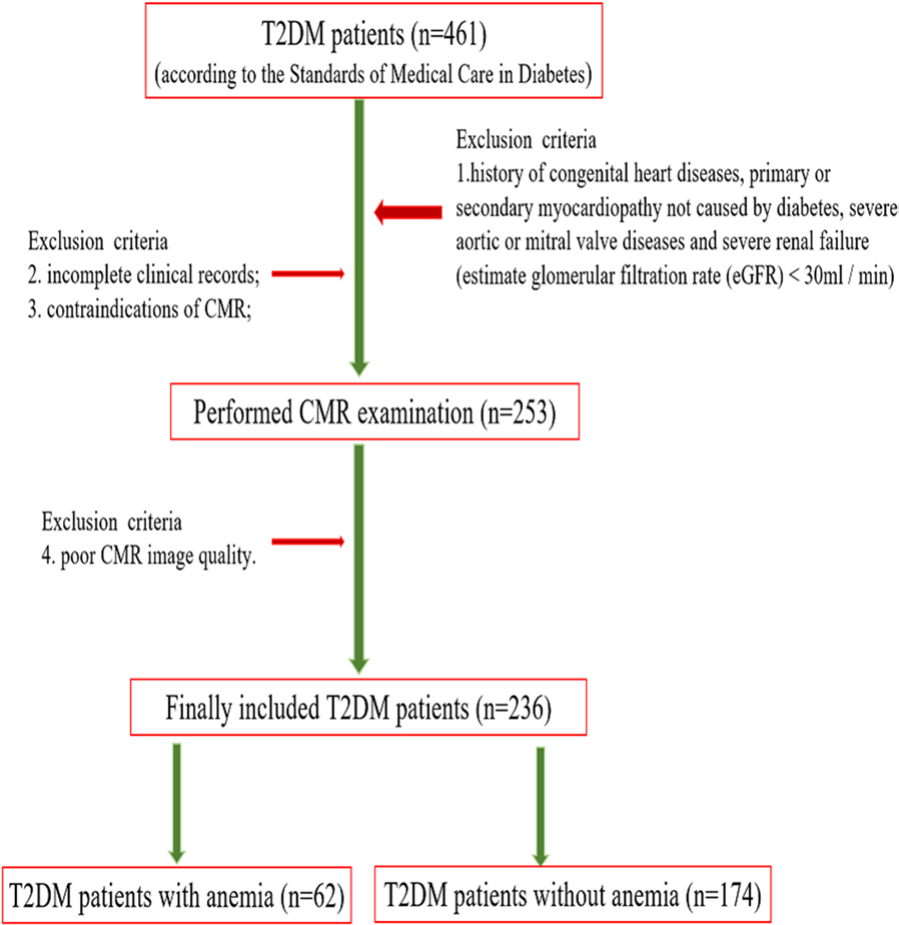
Flowchart of the cohort study. *CMR* cardiac magnetic resonance

Initially, 561 patients who were diagnosed with T2DM according to the Standards of Medical Care in Diabetes [16] and underwent CMR examination from September 2015 to June 2022 were retrospectively included in this study. The exclusion criteria were as follows: (1) patients with a history of congenital heart diseases, primary or secondary myocardiopathy not caused by T2DM, severe aortic or mitral valve diseases, and severe renal failure (estimated glomerular filtration rate (eGFR) < 30 ml/min); (2) incomplete clinical records; and (3) contraindications to CMR or poor CMR image quality. The inclusion criteria for the control group were as follows: (1) no T2DM or impaired fasting glucose; (2) no history of diseases that could impair cardiac function, such as coronary heart disease, hypertension, valvular heart disease, cardiomyopathy, systemic diseases and so on; and (3) normal cardiac function. Finally, 236 T2DM patients (122 males, 51.7%) and 67 controls (34 males, 50.7%) were included in this study. Among the T2DM group, patients were classified as having T2DM with anemia (n = 62, 33 males) or T2DM without anemia (n = 174, 89 males). The criteria for the diagnosis of anemia were consistent with the WHO criteria [17]. That is, for adults (except pregnant females), hemoglobin (Hb) concentration less than 120 g/l in females or 130 g/l in males would be diagnosed as anemia. A detailed enrollment flowchart is shown in Fig. 1.

### Basic information and laboratory data collection

Basic data, including sex, age, height, weight, systolic and diastolic blood pressure (SBP; DBP), heartbeat, and smoking history, were extracted from the medical records. Laboratory data, including Hb concentration, serum lipid level (total cholesterol, TC; triglyceride, TG; high-density lipoprotein, HDL; low-density lipoprotein cholesterol, LDL), eGFR and serum creatinine, were collected. In addition, for DM patients, extra data acquisition included glycated hemoglobin (HbA1c), duration of diabetes (years), use of antidiabetic drugs (α-glucosidase inhibitors, biguanides, sulfonylureas, glucagon-like peptide-1/dipeptidyl peptidase-4 inhibitors, sodium-glucose cotransporter 2 inhibitors, and insulin) and complications (nephropathy, retinopathy, peripheral vascular disease, neuropathy).

### CMR examination protocol

All enrolled patients underwent CMR examinations by two types of 3.0-T whole body scanners (MAGNETOM Skyra and MAGNETOM Trio Tim; Siemens Medical Solutions, Erlangen, Germany) with a 32-channel body phased-array coil in the supine position. To obtain better images, a standard ECG-triggering device was used, and data were collected during a breath-hold. A steady-state free precession (SSFP) sequence was used to obtain cine images of LV short-axis views and long-axis views (including four-chamber, three-chamber and two-chamber views) with the following parameters: temporal time, 39.34/40.35 ms; echo time, 1.22/1.20 ms; field of view, 234 × 280/250 × 300 mm^2^; slice thickness, 8.0 mm; flip angle, 39°/50°; and matrix size, 208 × 139/192 × 162 pixels.

### Image analysis

LV volume and functional parameters were acquired by two experienced radiologists who had at least 3 years of CMR experience and were blinded to patients’ clinical data by using offline and commercial software (cvi42, v.5.11.2; Circle Cardiovascular Imaging, Inc., Calgary, AB, Canada). The endocardium and epicardium of the LV end systolic phase and LV end diastolic phase on the short axis were carefully manually delineated layer by layer from the apex to the bottom of the heart to obtain LV function parameters, including LV end-diastolic volume index (LVEDVI), end-systolic volume index (LVESVI), stroke volume index (LVSVI), ejection fraction (LVEF), and LV mass index (LVMI) [18]. The papillary muscles and trabeculae were included in the LV cavity parameters and excluded from the LVMI. The LV concentricity index (LVCI) was calculated as LVM/LVEDV [19]. The endocardium and epicardium of the short-axis (all layers), 4-chamber long-axis (one layer), 3-chamber long-axis (one layer) and 2-chamber long-axis (one layer) cine slices were manually drawn at end-diastole to analyze the LV global strain parameters, including LV global radial peak strain (GRPS), global circumferential peak strain (GCPS), and global longitudinal peak strain (GLPS). 3D myocardial strains were used in this study. Due to the contractile nature of the heart, GLPS and GCPS are negative, while GRPS is positive [20].

### Reproducibility

Forty random patients, including 10 controls and 30 T2DM patients, were assessed to verify the intraobserver and interobserver variabilities. The intraobserver variability was tested by two sets of data obtained by the same observer (observer 1), 1 month apart. The data from observer 2 (who was blinded to all patient information and the results of observer 1) and observer 1 were used to verify the intraobserver variability.

### Statistical analysis

The Shapiro‒Wilk test was used to test for the distribution of continuous data. Normally distributed continuous data are expressed as the mean ± standard deviation, and nonnormally distributed continuous data are presented as the median (25–75% interquartile range). One-way analysis of variance (ANOVA) with Bonferroni’s or Tamhane’s T2 post hoc correction and the Kruskal‒Wallis test were used to compare normally distributed data and nonnormally distributed data among controls and T2DM patients with and without anemia, respectively. Independent T tests and the Mann‒Whitney U test were used to compare two groups of continuous data. Categorical variables are presented as frequencies (percentages) and were analyzed using the chi-square test. Pearson’s and Spearman correlation coefficients were used to determine the correlation between LV global strains and clinical indices, such as sex, age, BMI, heart rate, SBP, DBP, T2DM duration, HbA1c, Hb, eGFR, TG, TC, HDL and LDL. Multivariable linear regression analysis was used to determine the predictors of LV global strain indices in all T2DM patients. Inter- and intraobserver agreements were determined by the evaluation of intraclass correlation coefficients (ICCs). SPSS version 25 (IBM, Armonk, New York, USA) was used to perform all analyses, and a two-tailed p < 0.05 was considered indicative of significance. GraphPad Prism software (version 9.0.0 (121), GraphPad Software Inc., San Diego, CA, USA) was used to draw the scatter plot of the correlation between Hb and PS.

## Results

### Baseline characteristics

Finally, 303 participants, including 67 controls, 62 T2DM with anemia patients and 174 T2DM without anemia patients, were enrolled. Among the three groups, the T2DM with anemia group was older than the control group and the T2DM without anemia group; Hb (107 g/l ± 12 vs. 142 g/l ± 13 vs. 139 g/l ± 12) and eGFR were significantly lower in the T2DM with anemia group than in the other two groups, while for the above three indices, the T2DM without anemia and the control groups did not show significant differences (all P < 0.001). The BMI and DBP of the T2DM without anemia group were higher than those of the control group and T2DM with anemia group, while the latter two groups did not show significant differences (p = 0.038 and 0.037); the HR and SBP of all T2DM patients were significantly higher than those of controls (p = 0.016 and < 0.001). The diabetes duration, HbA1c and medications were not significantly different between T2DM patients with or without anemia (all p > 0.05). Nearly all complications presenting in the T2DM with anemia group and the T2DM without anemia group showed no significant difference between the groups except for nephropathy (29.03% vs. 9.20%, p < 0.001). The detailed basic information is shown in Table 1.

**Table 1:** Baseline characteristics of the study population

	Controls (n = 67)	T2DM without anemia (n = 174)	T2DM with anemia (n = 62)	P value
Demographics				
Male, n (%)	34 (50.7)	89(51.1)	33 (53.2)	0.952
Age, years	56.73 ± 9.66	57.07 ± 10.51	63.29 ± 12.23*^§^	0.000
BMI (kg/m^2^)	23.41 ± 3.06	24.54 ± 2.95*	24.23 ± 3.34	0.038
Heart rate (beats/min)	74 ± 10	80 ± 16*	81 ± 15*	0.016
SBP (mmHg)	118 ± 12	130 ± 18*	129 ± 19*	0.000
DBP (mmHg)	74 ± 9	79 ± 13*	77 ± 13	0.037
Smoking history, n (%)	15 (22.38)	43 (24.70)	21 (33.87)	0.273
Diabetes duration, years	–	6 (3.0, 10.0)	5 (2.0, 9.0)	0.272
Laboratory data				
Hb, (g/L)	142 ± 13	139 ± 12	107 ± 12*^§^	0.000
HbA1c, %	–	7.41 ± 1.75	7.19 ± 1.17	0.280
TG (mmol/L)	1.28 (0.99, 1.65)	1.49 (1.01, 2.19)	1.25 (0.96, 1.71)*	0.042
TC (mmol/L)	4.81 (3.97, 5.24)	4.19 (3.33, 4.90)*	3.43 (2.99, 4.45)*^§^	0.000
HDL (mmol/L)	1.29 (1.12, 1.53)	1.17 (0.96, 1.42)*	1.14 (0.88, 1.39)*	0.001
LDL (mmol/L)	2.91 (2.24, 3.38)	2.26 (1.71, 2.94)*	1.74 (1.41, 2.37)*^§^	0.000
eGFR (mL/min/1.73 m2)	93.78 ± 15.13	87.53 ± 19.46	70.67 ± 22.37*^§^	0.000
Creatinine, umol/L	71.12 ± 15.43	76.15 ± 22.34*	94.06 ± 31.25*^§^	0.000
Complications, n (%)				
Nephropathy	–	16 (9.20)	18 (29.03)^§^	0.000
Retinopathy	–	13 (7.47)	4 (6.45)	1.000
Peripheral vascular disease	–	35 (20.11)	14 (27.42)	0.681
Neuropathy	–	33 (18.97)	6 (9.68)	0.091
Medications, n (%)				
Insulin	–	32 (18.39)	12 (19.35)	0.979
Biguanides	–	72 (41.38)	24 (38.71)	0.713
Sulfonylureas	–	31 (17.82)	6 (9.68)	0.130
α‑Glucosidase inhibitor	–	34 (19.54)	8(12.90)	0.241
Others	–	25 (14.37)	8 (12.90)	0.775
No	–	37 (21.26)	11 (17.74)	0.554

Data are presented as the mean ± SD, median (Q1, Q3) or number (percentage)

*BMI* body mass index, *SBP* systolic blood pressure, *DBP* diastolic blood pressure, *Hb* hemoglobin, *TC* total cholesterol, *TG* triglyceride, *HDL* high-density lipoprotein, *LDL* low-density lipoprotein cholesterol, *eGFR* estimate glomerular filtration rate

^*^P less than 0.017 vs. controls

^§^P less than 0.05 vs. T2DM patients without anemia

### Comparison of LV geometric and functional parameters among the three groups

**Fig. 2:**
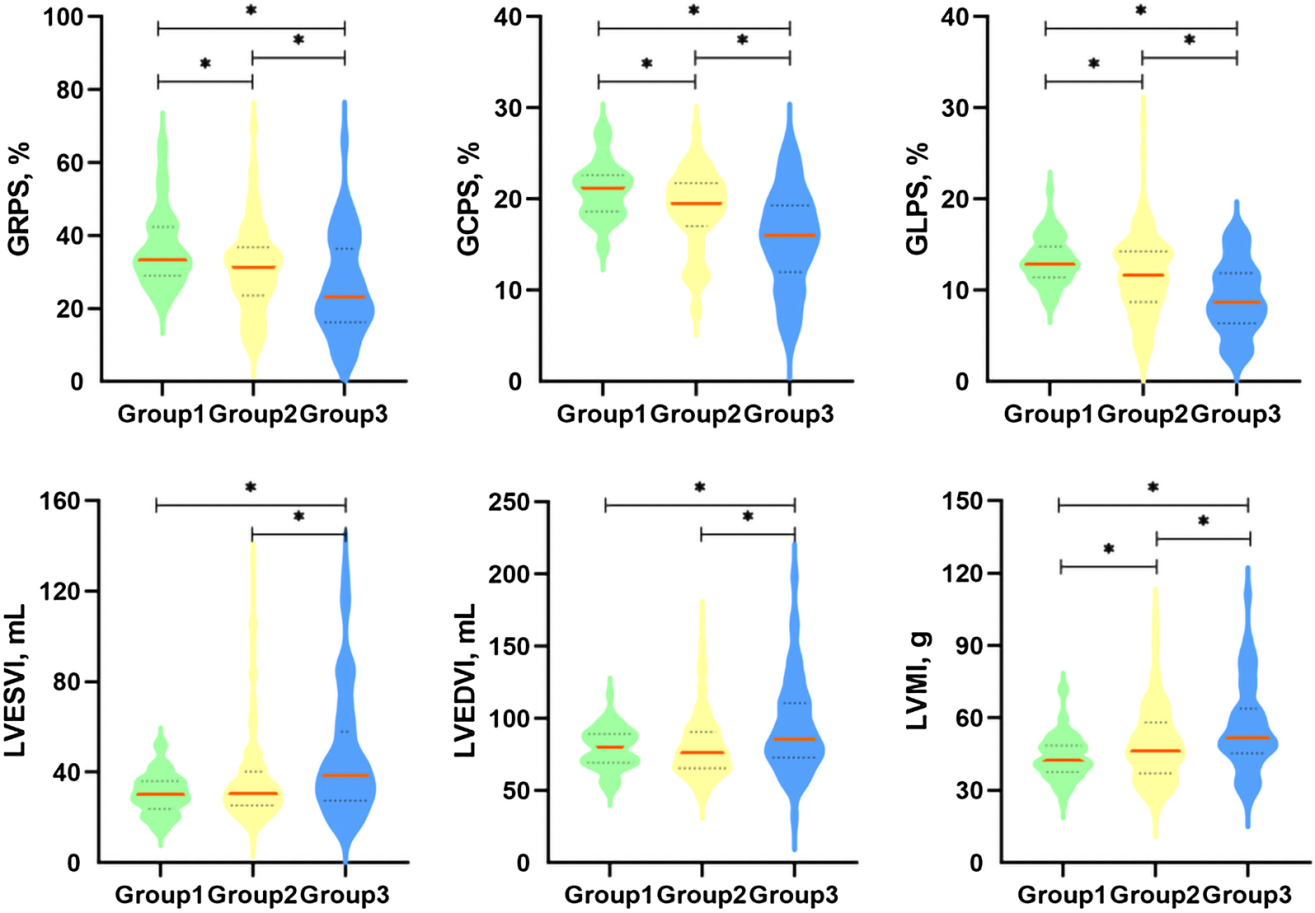
Comparison of three subgroups of LV global peak strains and LV function parameters. * means P less than 0.017. Group1 represents normal controls; Group 2 represents T2DM patients without anemia; Group3 represents T2DM patients with anemia. *GRPS* global radial peak strain, *GCPS* global circumferential peak strain, *GLPS* global longitudinal peak strain, *LV* left ventricular, *EDV* end diastolic volume, *ESV* end systolic volume, *M* mass, *I* indexed to BSA

For basic LV geometric and functional parameters, the LVEDVI and LVESVI of the T2DM with anemia group were significantly higher than those of the control group and the T2DM without anemia group, while the latter two groups showed no significant difference (p < 0.05). The LVSVI of the T2DM with anemia group did not show a significant difference compared with the control group and the T2DM without anemia group, while the control group had a higher LVSVI than the T2DM without anemia group (p = 0.025). The control group had a higher mean LVEF than the two T2DM groups, while the T2DM without anemia group had a similar mean LVEF than the T2DM with anemia group (61.6% ± 7.8 vs. 55.6% ± 11.2 vs. 51.9% ± 12.0, p < 0.001). The LVMI progressively and significantly increased from controls, through T2DM patients without anemia, to T2DM patients with anemia (43.7 g ± 9.0 vs. 48.8 g ± 15.2 vs. 55.5 g ± 17.4, P < 0.001. Figure 2), while LV concentricity indices were not significantly different among the three groups (p = 0.096). The detailed information is shown in Table 2.

**Table 2:** Comparisons of CMR derived LV function and LV global strains findings among controls, T2DM patients without anemia and T2DM patients with anemia groups

	Controls (n = 67)	T2DM without anemia (n = 174)	T2DM with anemia (n = 62)	P value
LV function parameters				
LVEDVI, mL	78.9 ± 13.7	80.7 ± 22.4	92.7 ± 30.9*^§^	0.001
LVESVI, mL	30.3 (23.8, 36.1)	30.6 (25.4, 40.3)	38.5 (27.4, 57.9)*^§^	0.000
LVSVI, mL	48.4 ± 9.0	44.3 ± 10.4*	46.9 ± 14.3	0.025
LVEF, %	61.6 ± 7.8	55.6 ± 11.2*	51.9 ± 12.0*	0.000
LVMI, g	43.7 ± 9.0	48.8 ± 15.2*	55.5 ± 17.4*^§^	0.000
LV concentricity index, g/mL	0.55 (0.48, 0.63)	0.59 (0.51, 0.70)	0.60 (0.48, 0.70)	0.096
Peak strain (%)				
Radial	33.4 (29.0, 42.4)	31.2 (23.6, 36.7)*	23.2 (16.2, 36.4)*^§^	0.000
Circumferential	−21.1 ± 3.0	−18.8 ± 4.3*	−15.6 ± 5.3*^§^	0.000
Longitudinal	−13.2 ± 2.5	−11.5 ± 4.3*	−8.9 ± 4.4*^§^	0.000
PSSR (1/s)				
Radial	1.9 (1.5, 2.7)	1.7 (1.2, 2.1)*	1.5 (0.9, 2.2)*	0.007
Circumferential	−1.0 (−1.2, −0.9)	−1.0 (−1.1, −0.8)*	−0.9 (−1.1, −0.7)*	0.001
Longitudinal	−0.7 (−0.8, −0.6)	−0.7 (−0.8, −0.5)*	−0.6 (−0.7, −0.4)	0.024
PDSR(1/s)				
Radial	−2.3 (−2.9, −1.8)	−1.9 (−2.6, −1.4)*	−1.4 (−2.2, −0.9)*^§^	0.000
Circumferential	1.2 ± 0.3	1.0 ± 0.4*	0.9 ± 0.3*^§^	0.000
Longitudinal	0.8 (0.6, 0.9)	0.7 (0.5, 0.9)	0.5 (0.4, 0.8)*^§^	0.000

Data are presented as the mean ± SD or median (Q1, Q3)

*T2DM* type 2 diabetes diabetes mellitus, *LV* left ventricular, *EDV* end diastolic volume, *ESV* end systolic volume, *SV* stroke volume, *EF* ejection fraction, *M* mass, *I* indexed to BSA, *PSSR* peak systolic strain rate, *PDSR* peak diastolic strain rate

“ − ” indicates the direction of strains (negative). Negative strain means shortening, thinning, and/or contraction, while positive strain means lengthening, thickening, and/or relaxation from end-diastole to end-systole

^*^P less than 0.05 vs. controls

^§^P less than 0.05 vs. T2DM patients without anemia

### Comparison of cardiac magnetic resonance-derived LV global strain among the three groups

**Fig. 3:**
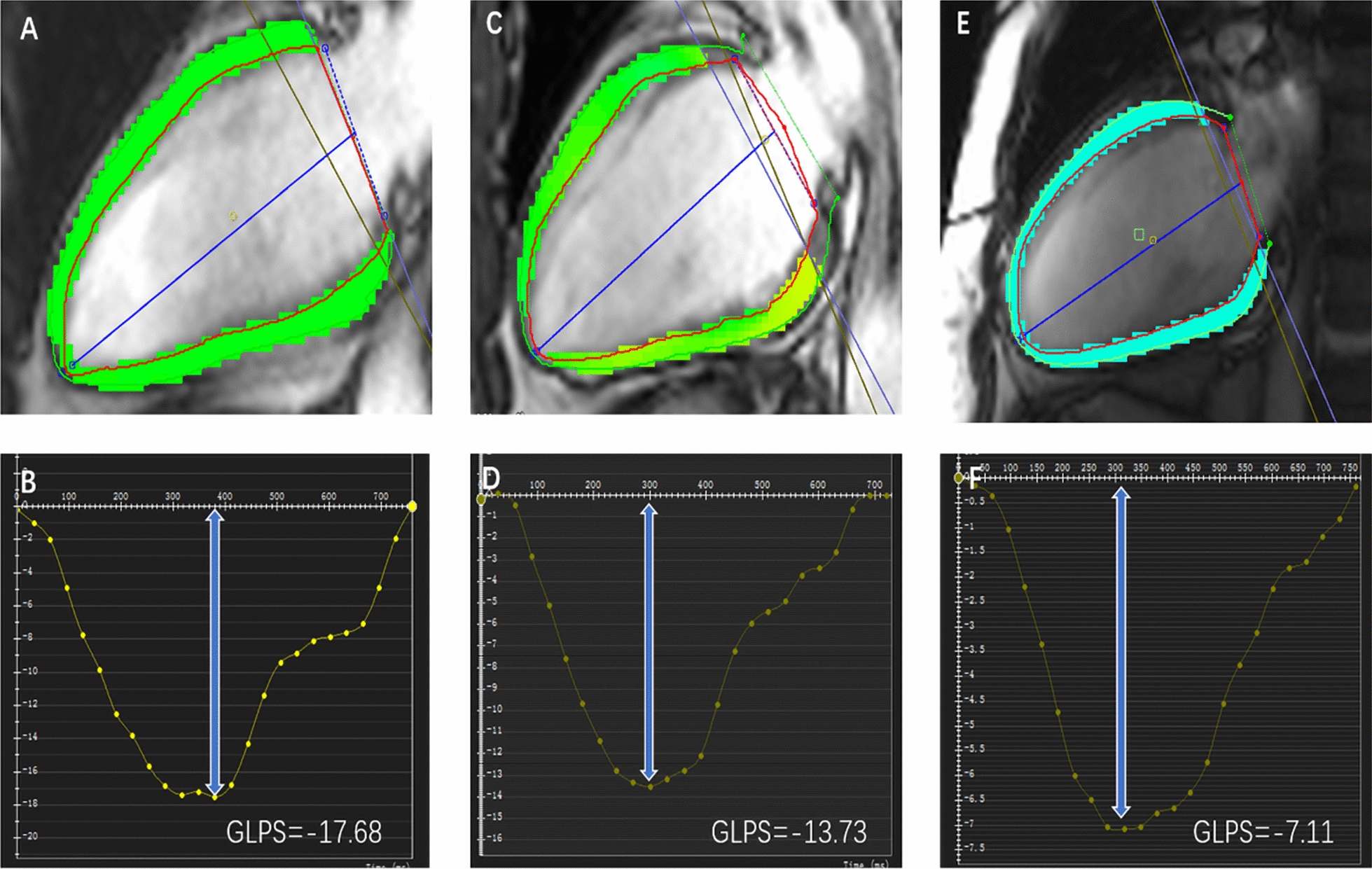
Representative CMR pseudocolor images at the end-diastole and CMR- derived peak strain curves. **A**, **C**, **E**: LV pseudocolor images in the vertical 2—chamber long‑axis; **B**, **D**, **F**: LV global peak strain curve in the longitudinal direction; **A**, **B**: a patient of control group; **C**, **D**: a T2DM patient without anemia; **E**, **F**: a T2DM patient with anemia. GLPS, global longitudinal peak strain

Among the three groups, all three directions of LV global peak strain progressively and significantly worsened from controls, through T2DM patients without anemia, to T2DM patients with anemia (radial 33.4% (29.0, 42.4) vs. 31.2% (23.6, 36.7) vs. 23.2% (16.2, 36.4); circumferential −21.1% ± 3.0 vs. −18.8% ± 4.3 vs. −15.6% ± 5.3; longitudinal −13.2% ± 2.5 vs. −11.5% ± 4.3 vs. −8.9% ± 4.4; all p < 0.001. Figure 2). Figure 3 shows representative CMR-derived longitudinal peak strain curves in a normal control, a T2DM patient without anemia, and a T2DM patient with anemia. For peak diastolic strain rate (PDSR), radial and circumferential PDSR progressively and significantly worsened from controls to T2DM patients without anemia to T2DM patients with anemia (all p < 0.001). The longitudinal PDSR of T2DM patients with anemia was significantly lower than that of the other two groups, while the other two groups showed no significant difference (p < 0.001). For peak systolic strain rate (PSSR), there were no significant differences between the T2DM with and without anemia groups in all three directions, while the control group had a higher PSSR than the two T2DM groups except for longitudinal PSSR (all p < 0.05, Table 2)**.**

### Associations between clinical parameters and LV global strains in all T2DM patients

**Fig. 4:**
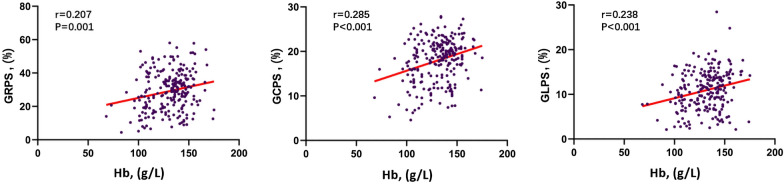
Linear regression analysis between the magnitude of LV peak strains and hemoglobin. *GRPS* global radial peak strain, *GCPS* global circumferential peak strain, *GLPS* global longitudinal peak strain, *Hb* hemoglobin

After univariate linear regression analysis, sex, HbA1c, Hb, eGFR, TC, HDL, and LDL were found to be significantly associated with all three directions of LV global PS (all p < 0.1) (Fig. 4). SBP was significantly associated with GRPS (p = 0.010) and GLPS (p = 0.008) but not with GCPS (p = 0.802). In addition, BMI, DBP, and TG were independently related to GLPS (all p < 0.1). Multivariate linear regression analysis revealed that sex, eGFR and Hb were independently associated with PS in all directions (all p < 0.05); HbA1c was associated with GRPS (β = −0.162, p = 0.013) and GCPS (β = −0.168, p = 0.007) but not with GLPS (p = 0.630). For GLPS, BMI had a negative influence (β = −0.161, p = 0.011), and HDL had a positive influence (β = 0.202, p = 0.023). Moreover, among all these indices, Hb concentration had the greatest effect on left ventricular GRPS (β = 0.237, p < 0.001), GCPS (β = 0.326, p < 0.001) and GLPS (β = 0.265, p < 0.001). The detailed information is shown in Table 3.

**Table 3:** Associations between clinical parameters and LV global strains in all T2DM patients

	GRPS				GCPS				GLPS			
	Univariable		Multivariable		Univariable		Multivariable		Univariable		Multivariable	
	β	P value	β	P value	β	P value	β	P value	β	P value	β	P value
Sex	0.170	0.009*	0.202	0.004*	0.191	0.003*	0.253	0.000*	0.231	0.000*	0.222	0.001*
Age	0.039	0.552			0.009	0.894			−0.037	0.568		
BMI	−0.057	0.391			−0.073	0.269			−0.191	0.004*	−0.161	0.011*
Heart rate	−0.005	0.934			−0.028	0.675			0.008	0.900		
SBP	−0.109	0.100*	−0.092	0.154	−0.017	0.802			−0.175	0.008*	0.106	0.105
DBP	−0.038	0.573			−0.001	0.983			−0.141	0.033*	0.067	0.314
Diabetes duration	0.101	0.187			0.059	0.444			0.100	0.191		
HbA1c	−0.233	0.000*	−0.162	0.013*	−0.236	0.000*	−0.168	0.007*	−0.177	0.080*	−0.030	0.630
Hb	0.207	0.001*	0.237	0.001*	0.285	0.000*	0.326	0.000*	0.238	0.000*	0.265	0.000*
eGFR	0.235	0.000*	0.149	0.036*	0.277	0.000*	0.143	0.033*	0.282	0.000*	0.136	0.044*
TG	−0.039	0.554			−0.006	0.922			−0.169	0.010*	0.008	0.931
TC	0.194	0.003*	0.078	0.475	0.241	0.000*	0.028	0.789	0.191	0.003*	0.036	0.793
HDL	0.134	0.041*	0.094	0.194	0.178	0.006*	0.046	0.507	0.254	0.000*	0.202	0.023*
LDL	0.134	0.041*	−0.125	0.236	0.178	0.006*	−0.019	0.854	0.254	0.000*	−0.120	0.289

Abbreviation of BMI, SBP, DBP, Hb, eGFR, TG, TC, HDL, LDL are shown in Table 1. *GRPS* global radial peak strain, *GCPS* global circumferential peak strain, *GLPS* global longitudinal peak strain

^*^ P less than 0.05

### Intraobserver and interobserver variability

The intraobserver and interobserver correlation coefficients were considered excellent. The ICCs of PS in all three directions were higher than 0.8, and the detailed information is summarized in Table 4.

**Table 4:** Intraobserver and interobserver variability

	Intra-observer		Inter-observer	
	ICCs	95%CI	ICCs	95%CI
GRPS	0.886	0.792–0.938	0.877	0.776–0.934
GCPS	0.941	0.891–0.968	0.921	0.855–0.957
GLPS	0.872	0.771–0.930	0.830	0.701–0.907

Abbreviation of GRPS, GCPS, GLPS are shown in Table 3

## Discussion

In this study, the combined effect of T2DM and hemoglobin concentration was investigated. The main findings were as follows: (1) Compared with the control group, T2DM patients had worse LV global strain and LVEF. (2) T2DM patients with anemia had the lowest LV global strain and highest LV mass, while the LV concentricity index did not show a significant difference compared to the controls and the T2DM patients without anemia. (3) Among all clinical indices, Hb was independently associated with LV global strain and was the main influencing factor of the LV global strain after adjustment for several factors in this study group.

Among the three groups in our study, T2DM patients with or without anemia had a lower LVEF, higher LVMI and worse LV global peak strain than the controls. The result of LVEF and LVMI is similar to a previous study [20]. T2DM leads to cardiac stiffness, myocardial fibrosis and hypertrophy by multiple mechanisms, such as hyperglycemia, systemic and cardiac insulin resistance, calcium disturbance in cardiomyocytes, inflammation, microvascular dysfunction and so on [21, 22], which put tremendous stress on the heart independently to other risk factors [23, 24]. LV global strain is acquired by tracking the myocardial motions between the epicardial and endocardial borders. It reflects the LV motion abnormalities early and sensitively [25]. LV global peak strains in three directions were significantly reduced from the controls to T2DM patients without anemia to T2DM patients with anemia, while the mean LVEF of all T2DM patients enrolled was more than 50% in our study. This reveals the fact that the damage due to T2DM and anemia was already present even when the LVEF was at a relatively normal level.

Although T2DM patients with anemia had highest LVM than the controls and the T2DM patients without anemia, the LVEDV in the T2DM with anemia group was also highest among all three groups in this study population, which led to the result that the LV concentricity index (calculated as LVM/LVEDV) was not significantly different from that in the other two groups. When patients have anemia, the body undergoes nonhemodynamic and hemodynamic changes to compensate for the insufficient oxygen supply. The main nonhemodynamic change involves increased erythropoiesis. The main hemodynamic change is the increased cardiac output caused by lower afterload, increased preload and positive inotropic and chronotropic effects. Gradually, cardiac cavity enlargement (increased LVEDV and LVESV) and LV hypertrophy (increased LV mass) develop [4]. This type of morphological change of the heart is known as eccentric hypertrophy [26], which is helpful to explain the abovementioned result that T2DM patients with anemia had the highest LVEDV and LVM.

As explained above, anemia and DM can each damage the heart in their own ways. Besides, some studies also have found when the two diseases are combined, cardiac function could be further impaired [9, 27]. This is similar with our results that some LV function parameters and all LV global peak strain of T2DM patients with anemia were worst among the three groups. T2DM patients could be more likely to suffer from anemia [28]. However, anemia in T2DM patients is likely to be unrecognized and lead to inadequate treatment [14, 29]. If anemia could be reversed in a timely manner in patients without overt heart disease, LV hypertrophy might be corrected [30, 31]. However, when anemia is severe or accompanied by other heart diseases, irreversible impairment would be left and the prognosis might be worse [32].Thus, once a diagnosis of T2DM is made, clinicians and patients should pay attention to detecting, preventing and managing anemia.

As was shown in this study, after multivariate linear regression analysis, eGFR and Hb concentration were found to be independently associated with all LV global peak strains. The reasons leading to anemia are complex and varied. In T2DM patients, many studies have focused on diabetic or nondiabetic chronic kidney diseases [33, 34]. However, a previous study also found that even when eGFR is > 60 mL/min/1.73 m^2^, T2DM patients were more likely to have anemia [35]. Kidney diseases are not the only reason for the decrease of Hb. Other risk factors could contribute to the decrease of Hb, such as iron deficiency, RBC loss, reduced RBC survival, inflammation and resistance to erythropoietin [28]. This might help to explain our results that when taking eGFR into consideration, the reduction in Hb concentration was also an independent risk factor for the deterioration of LV global strain. Elderly people are susceptible to anemia [36]. Our results shows that T2DM patients with anemia were older than participants in the other two groups in this study population.

### Limitations

This study has some limitations. First, owing to the nature of the retrospective and single-center study, there might be some selection bias. Second, the final outcomes of the T2DM patients, which might be useful to further understand the influence of anemia, were not tracked because of the nature of the cross-sectional study. Last, most of our patients had mild to moderate anemia, and few had severe anemia. Thus, the effect of the grades of anemia severity on the heart was not explored. In the future, we will increase the sample size to more accurately investigate the impact of anemia on the heart.

## Conclusion

Anemia has additive deleterious effects on LV function and LV global strains of T2DM patients. The Hb concentration is an independent factor in LV global strains. Anemia should be given more attention when patients are diagnosed with T2DM, and regular detection and proper prevention of anemia might be beneficial for T2DM patients.

## Data Availability

The datasets generated during and/or analyzed in the current study are available from the corresponding author upon reasonable request.

## Abbreviations

CMR: Cardiac magnetic resonance
DBP: Diastolic blood pressure
eGFR: Estimated glomerular filtration rate
GCPS: Global circumferential peak strain
GRPS: Global radial peak strain
GLPS: Global longitudinal peak strain
HDL: High-density lipoprotein
Hb: Hemoglobin
ICCs: Intraclass correlation coefficients
IDF: International Diabetes Federation
LV: Left ventricular
LVCI: Left ventricular concentricity index
LDL: Low-density lipoprotein cholesterol
LVEDVI: Left ventricular end-diastolic volume index
LVEF: Left ventricular ejection fraction
LVESVI: Left ventricular end-systolic volume index
LVMI: Left ventricular mass index
LVSVI: Left ventricular stroke volume index
RBC: Red blood cell
SBP: Systolic blood pressure
SV: Stroke volume
T2DM: Type 2 diabetes mellitus
TC: Total cholesterol
TG: Triglyceride
